# Expression and activation of toll-like receptor 3 and toll-like receptor 4 on human corneal epithelial and conjunctival fibroblasts

**DOI:** 10.1186/1476-9255-11-3

**Published:** 2014-02-04

**Authors:** Nir Erdinest, Gal Aviel, Eli Moallem, Irene Anteby, Claudia Yahalom, Hadas Mechoulam, Haim Ovadia, Abraham Solomon

**Affiliations:** 1 Department of Ophthalmology, Hadassah-Hebrew University Medical Center, Jerusalem, Israel; 2 Clinical Immunology and Allergy Unit, Hadassah-Hebrew University Medical Center, Jerusalem, Israel; 3 Department of Neurology, The Agnes Ginges Center for Human Neurogenetics, Hadassah-Hebrew University Medical Center, Jerusalem, Israel; 4 Cornea & Refractive Surgery Service, Department of Ophthalmology, Hadassah-Hebrew University Medical Center, Jerusalem 91120, Israel

**Keywords:** Toll-like receptor, Corneal epithelial, Conjunctival fibroblasts, Lipopolysaccharide, Polyinosinic:polycytidylic acid, Culture cells

## Abstract

**Background:**

Toll-like receptors (TLRs) are recognized as important contributors to the initiation and modulation of the inflammatory response in the eye. This study investigated the precise expression patterns and functionality of TLRs in human corneal epithelial cells (HCE) and in conjunctival fibroblasts (HCF).

**Methods:**

The cell surface expression of TLRs 2-4, TLR7 and TLR9 in HCE and HCF was examined by flow cytometry with or without stimulation with lipopolysaccharide (LPS) or polyinosinic:polycytidylic acid (poly I:C). The mRNA expression of the TLRs was determined by real-time PCR. The protein content levels of interleukin (IL)-6, IL-8, IL-1β and tumor necrosis factor-α (TNF-α) were measured in HCE and HCF using multiplex fluorescent bead immunoassay (FBI).

**Results:**

The surface expression of TLR3 and TLR4 was detected on both HCE and HCF. Following incubation with LPS, the percentage of HCE cells staining for TLR4 decreased from 10.18% to 0.62% (P < 0.001). Incubation with poly I:C lowered the percentage of HCE cells positive for TLR3 from 10.44% to 2.84% (P < 0.001). The mRNA expression of TLRs2, 4, 7 and 9 was detected in HCE only. Activation of HCE with LPS complex elicited protein secretion up to 4.51 ± 0.85-fold higher levels of IL-6 (P < 0.05), 2.5 ± 0.36-fold IL-8 (P > 0.05), 4.35 ± 1.12-fold IL-1β (P > 0.05) and 29.35 ± 2.3-fold TNFα (P < 0.05) compared to cells incubated in medium.

**Conclusions:**

HCF and HCE both express TLRs that respond to specific ligands by increasing cytokine expression. Following activation, the surface expression of TLR3 and TLR4 on HCE is decreased, thus creating a negative feedback loop, mitigating the effect of TLR activation.

## Background

The ocular surface consists of the eyelid, cornea, conjunctiva, lacrimal gland and tear film, and provides the first line of defense against mechanical and biological insults [1]. The corneal epithelium, in particular, serves a critical function of the mucosal innate immune system, as it is constantly exposed to microorganisms and their virulent products [2]. An important characteristic of the corneal epithelial cell function is the ability to recognize a wide range of pathogens, and upon challenge to secrete cytokines and other immune mediators, thereby initiating an efficient, highly sensitive, immune response [3].

Microorganisms possess highly conserved motifs and pathogen associated molecular patterns (PAMP) that are recognized by pattern recognition receptors (PRR) found on cells of the innate immune system. Toll-like receptors (TLR) are a family of PRR, capable of recognizing and responding to various PAMP. Upon stimulation of the receptor, a cascade of intracellular signaling is initiated, culminating in the activation of NF-κB and secretion of a variety of cytokines, chemokines and expression of co-stimulatory molecules. To date, ten different functional TLRs were demonstrated in humans, each presents a tendency towards a specific PAMP. For example, TLR4 recognizes lipopolysaccharide (LPS), the endotoxin of Gram-negative bacteria, while TLR3 is a sensor of viral dsRNA [4].

A widespread expression of various TLRs has been demonstrated in the human eye, including the retina, uvea, lacrimal gland and conjunctiva [5,6]. TLRs are also found in the human cornea, and have been implicated in several infectious diseases [7]. Despite the importance of the TLR family, the exact localization in the human cornea of its various members and their ability to respond to antigenic stimuli is yet to be fully understood. Moreover, there are discrepancies in the literature regarding the characterization of TLR3 and TLR4 in HCE cells.

Like HCE cells, conjunctival fibroblasts have long been implicated in the pathophysiology of several chronic disorders of the ocular surface such as ocular cicatricial pemphigoid (OCP), dry eye, pterygium and vernal keratoconjunctivitis [6-8]. Unlike HCE cells that have been thoroughly investigated, this cell population did not receive similar attention. We believe that characterizing the TLR family in these cells might help future research to elucidate the nature of some diseases of the ocular surface.

In the present study, we present the results of a systematic approach to the characterization of TLR3 and TLR4 in primary HCE and HCF.

## Methods

The Hadassah Medical Center Institutional Review Board (IRB) approval was obtained for this study (IRB protocol number and version: EFA-EFE-IV-01). This study followed the tenets of the Declaration of Helsinki.

### Human corneal epithelial (HCE) cells

HCE cells were cultured from human corneoscleral rim explants, taken from several different human donors, provided by the Department of Ophthalmology at the Hadassah Medical Center, using a previously described method [9].

HCE cells were cultured in supplemented hormonal epithelial medium (SHEM) [10] and were incubated at 37°C under 95% humidity and 5% CO_2_. The culture medium was replaced every other day. Cultures were kept for 10 to 14 days until a density of 90% confluence was observed.

### Human conjunctival fibroblasts (HCF)

Human conjunctiva explant cultures were established using specimens obtained at the time of strabismus surgery and were used for the isolation and culture within 1–3 hours after surgery. HCF were cultured as previously described [11]. In brief, HCF were cultured in supplemented fibroblasts medium which contained Dulbecco’s modified Eagle medium (DMEM) with nutrient mixture F12 (Gibco), supplemented with 4 mM glutamine, 10% fetal calf serum (FCS), 100 U of penicillin and 100 μg of streptomycin per ml. The HCF were incubated at 37°C under 95% humidity and 5% CO_2_. The medium was replaced every 2–3 days. Cultures were kept for 10 to 14 days until a density of 90% confluence was observed.

### Human neuron-committed teratocarcinoma (NT2) cells

Human neuron-committed teratocarcinoma (NT2) cells line do not express TLRs and thus served as negative control for the flow cytometry and PCR analysis. NT2 cells line was grown in DMEM with nutrient mixture F12 (Gibco), supplemented with 4 mM glutamine, 10% fetal calf serum (FCS), 100 U of penicillin and 100 μg of streptomycin per ml. Briefly, 10^6^ NT2 cells were seeded into 75-cm^2^ flasks with 5 ml of medium.

### Human peripheral blood mononuclear (HPBM) cells

Mononuclear cells are known to express various TLRs, and thus served as positive control for the flow cytometry and PCR analysis. The cells were separated from outdated venous blood samples obtained from the Hadassah Medical Center Blood Bank. Blood samples were placed in 50 ml polypropylene tubes and mixed with equal volumes of PBS. Ficoll-Hypaque solution (Sigma Chemical Co. St. Louis) was slowly layered under the blood/PBS mixture. Afterwards, the 50 ml tube was centrifuged 30 min at 2000 rpm (900 g) with no brake. The mononuclear cell layer was aspirated using a sterile pipette and transferred into a second centrifuge tube.

### Monoclonal antibodies

Anti-human monoclonal antibody to TLR3 (TLR3.7) was purchased from Hycult Biotech and anti-human monoclonal antibody to TLR4 (HTA125) was purchased from NOVUS Biologicals. FITC-conjugated AffiniPure donkey anti-mouse IgG (H + L) antibody and AffiniPure F(ab’)_2_ fragment donkey anti-human IgG (H + L) were purchased from Jackson ImmunoResearch Laboratories. Purified anti-mouse IgG1 isotype control was purchased from BioLegend.

### TLR3 and TLR4 expression in HCE cells - by flow cytometry

The cells were either incubated with TLR-specific antibodies or with isotype-matched control antibodies. We also used NT2 cells as a negative control and HPBM cells as a positive control.For flow cytometry analysis we used anti-human monoclonal antibodies to human TLR3 (TLR3.7) (Hycult Biotech) and TLR4 (HTA125) (NOVUS Biologicals). HCE cells were washed twice in FACS buffer (Dulbecco’s PBS containing 0.5% BSA and 0.1% sodium azide) and incubated with the indicated monoclonal antibodies (1 μg) together with human IgG (10 μg) for 30 min at 4°C. After the cells were washed twice with the above buffer, FITC-labeled secondary antibody was added and further incubated for 30 min at 4°C. The cells were then analyzed on a FACScan (BD Biosciences). For isotype control, the primary antibodies used were anti-mouse IgG1 or anti-mouse IgG2a.

For intracellular staining of TLR3 and TLR4, the cells were fixed in 1% formaldehyde and permeabilized using 0.1% saponin [12]. The cells were subsequently incubated with monoclonal antibodies and FITC-secondary antibodies, as described above. The antibodies were diluted using a 0.1% saponin-containing PBS solution.

### Expression of TLRs-specific mRNA in HCE cells by real-time PCR

Quantitative real-time reverse transcriptase polymerase chain reaction (RT-PCR) was conducted in triplicates in a final volume of 15 μL. The reaction containing 2× power SYBR green PCR Master Mix (Applied Biosystems, Warrington, UK), cDNA together with forward and reverse primer of TLRs 2–4, TLR7 and TLR9 (Table 1). The primers were selected with an AB Primer Express program (v.2.0, Applied Biosystems, USA) and synthesized (Syntezza, Israel). Amplification was performed using the ABI PRISM® 7000 sequence detector (Applied Biosystems,USA).

**Table 1:** Forward and reverse primers of TLRs 2–4, TLR7 and TLR9

**Gene**	**Primer**	**Sequence**
TLR2	Sense	5′-GCCAAAGTCTTGATTGATTGG-3′
	Antisense	5′-TTGAAGTTCTCCAGCTCCTG-3′
TLR3	Sense	5′-CGCCAACTTCACAAGGTA-3′
	Antisense	5′-GGAAGCCAAGCAAAGGAA-3′
TLR4	Sense	5′-TGGATACGTTTCCTTATAAG-3′
	Antisense	5′-GAAATGGAGGCACCCCTTC-3′
TLR7	Sense	5′-AGTGTCTAAAGAACCTGG-3′
	Antisense	5′-CCTGGCCTTACAGAAATG-3′
TLR9	Sense	5′-GTGCCCCACTTCTCCATG-3′
	Antisense	5′-GGCACAGTCATGATGTTGTTG-3′
HPRT1	Sense	5′-AGATGGTCAAGGTCGCAAGC-3′
	Antisense	3′-CATATCCTACAACAAACTTGTCTGGAA-5′

### HCE and HCF activation

For TLR activation, HCE and HCF cells were incubated for 4 hours with either LPS 1 μg/ml supplemented with 500 ng/ml Cluster of differentiation 14 (CD14) and 500 ng/ml Lipopolysaccharide binding protein (LBP), together defined as LPS complex as previously described [13,14] or with Poly I:C at a dose of 25 μg/ml [15]. For maximal induction of IL-6 and IL-8, the stimulus exposure time lasted 4 hours for protein content measurement and 3 hours for mRNA expression level measurement. For TNF-α and IL-1β the stimulus lasted 15 hours for protein content measurement, and 12 hours for mRNA expression level measurements.

### Cytokines protein secretion after stimulation of HCE and HCF with LPS and poly I:C - by a multiplex fluorescent bead immunoassay (FBI)

The cytokines protein concentration levels were measured using a multiplex fluorescent bead immunoassay (CBA, Human Inflammatory Cytokines Kit, BD Biosciences, USA). The test was performed and analyzed according to the manufacturer’s instructions and was performed as described previously [16].

### Cytokines mRNA expression in response to activation of HCE and HCF with LPS and poly I:C - by real-time polymerase chain reaction

Total RNA was extracted from the cells samples with RNAqueous Kit (Ambion, Austin, TX, USA) following the manufacturer’s instructions. cDNA was synthesized from purified and concentrated 0.5 μg RNA from each sample using a High Capacity cDNA Reverse Transcription Kit (Applied Biosystems, ABI, USA).

Real-time polymerase chain reaction (PCR) was performed using TaqMan® Gene Expression Assays (Applied Biosystems, Foster City, CA, USA) in the ABI Prism 7900HT Sequence Detection System (Applied Biosystems, Foster City, CA, USA) as described previously (18). Negative controls were included to evaluate DNA contamination of isolated RNA and reagents. The fold changes of the gene expression in the samples were normalized to the endogenous gene hypoxanthine phosphoribosyltransferase 1 (HPRT1; Applied Biosystems, Foster City, CA, USA).

### Statistical analysis

All tests were carried out on four independent cell cultures, and performed in triplicates for each of the treatments. Statistical analysis and multiple comparisons were performed by one-way ANOVA using the InStat software version 3.0 (InStat software Inc., USA).

## Results

### HCE cells express TLR3 and TLR4 on the cell surface

**Figure 1:**
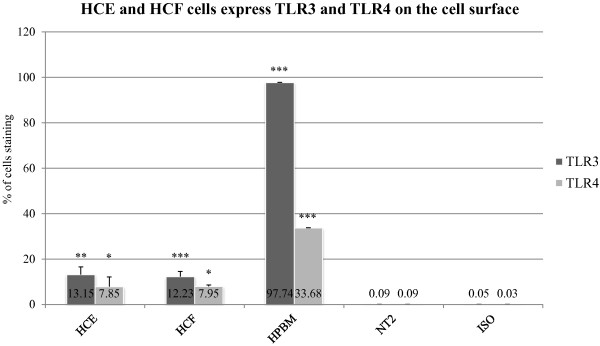
**HCE, HCF and HPBM cells expression of TLR3 and TLR4 on the cell surface, examined by flow cytometry.** NT2 cells served as a negative control. Cells were immunostained with TLR3-specific antibody, TLR4-specific antibody or isotype control antibody. Data shown (mean ± SD) are representative of four independent experiments. The single asterisk (p < 0.05), the double asterisks (p < 0.01) and the three asterisks (p < 0.001) represents statistical significance for experiments. TLR: Toll-like receptors, ISO: Isotype-control, NT2: Neuron-committed teratocarcinoma, HPBM: Human peripheral blood mononuclear, HCE: Human corneal epithelial cells.

Our aim was to determine whether HCE cells express TLR3 and TLR4 on their cell surface. Flow cytometry analysis of HCE cells using specific antibodies to TLR3 and TLR4 is presented in Figure 1. As shown, TLR3 (13.15 ± 3.43%; P < 0.01) as well as TLR4 (7.85 ± 4.32%; P < 0.05) were present on the cell surface of HCE cells and as compared with isotype control. HPBM cells were stained as well showing TLR4 on the cell surface (33.68 ± 0.15%; P < 0.001). In these cells TLR3 staining was positive only when cells were initially permeabilized with 0.1% saponin (97.74 ± 1.98%; P < 0.001).

### HCF cells express TLR3 and TLR4 on the cell surface

We found that both TLR3 and TLR4 were present on the cell surface of HCF cells (Figure 1) using flow cytometry analysis. As compared to isotype control, we found significant staining both for TLR3 (12.23 ± 2.36%; P < 0.001) and TLR4 (7.95 ± 0.7%; P < 0.001). The same controls were used as in the experiments with HCE cells, NT2 cells were minorly stained (0.09 ± 003; P > 00.5), HPBM cells were stained for TLR4 (33.68 ± 0.15%; P < 0.001) and for TLR3 (97.74 ± 1.98%; P < 0.001, Figure 1).

### HCE cells express TLR-specific mRNA

**Figure 2:**
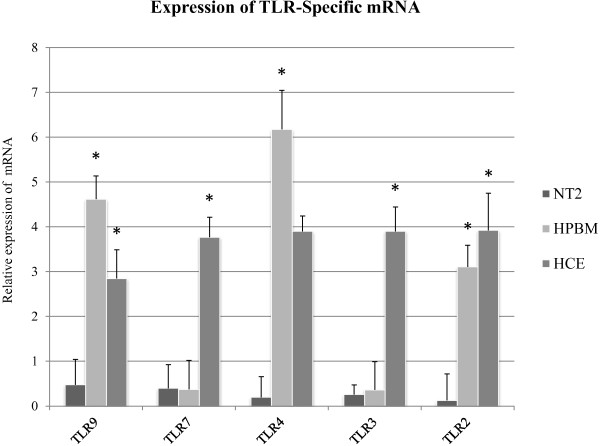
**Expression of TLRs-specific mRNA in HCE cells.** The mRNA expression of TLR 2–4, TLR7 and TLR9 was examined by Real-time PCR. TLRs 2–4, TLR7 and TLR9 mRNA expression in HCE cells is shown relative to HPRT1. Data derived from three independent experiments and shown as mean ± SD. The single asterisk (p < 0.05) represent statistical significance for experiments. TLR: Toll-like receptors, NT2: Neuron-committed teratocarcinoma, HPBM: Human peripheral blood mononuclear, HCE: Human corneal epithelium.

Real-time PCR results showed that specific mRNAs encoding for TLR2, TLR4, TLR7 and TLR9 were present in HCE cells. The mRNA expression of TLR2 was 3.92 ± 0.82 (P < 0.01) fold; TLR4 3.89 ± 0.34 (P < 0.01) fold; TLR7 3.76 ± 0.44 (P < 0.01) fold and TLR9 2.84 ± 0.63 (P < 0.01) fold compared to HPRT1 mRNA expression (Figure 2).

We have also shown the expression of these receptors in HPBM cells. The mRNA expression in HPBM cells of TLR2 was 3.1 ± 0.48 (P < 0.01) fold; TLR4 6.17 ± 0.86 (P < 0.01) fold and TLR9 4.61 ± 0.51 (P < 0.01) fold (Figure 2) and TLR 3 and TLR 7 were not expressed in HPBM cells. In NT2 none of these receptors was expressed (Figure 2).

### TLR3 and TLR4 surface expression decreases following activation in HCE cells

**Figure 3:**
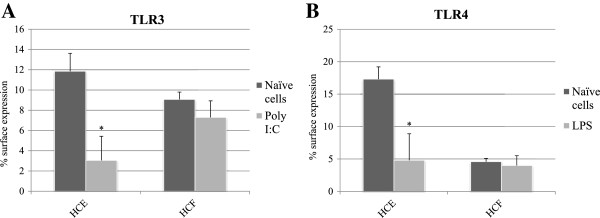
**TLR3 and TLR4 surface expression in HCE and HCF cells decreases following activation using poly I:C or LPS, respectively.** TLR3 surface expression in naïve, HCE and HCF cells following activation using poly I:C **(A)**. TLR4 surface expression in naïve, HCE and HCF cells following activation using LPS **(B)**. Data derived from three independent experiments and shown as mean ± SD. The asterisk (p < 0.05) represent statistical significance following activation. TLR: Toll-like receptors, HCE: Human corneal epithelium, LPS: Lipopolysaccharide, Poly I:C: polyinosinic:polycytidylic acid.

After determining that HCE express both TLR3- and TLR4-specific mRNA and express the receptors on the cell surface, we investigated whether stimulation by their respective ligands might affect the level of surface expression. For activation we used poly I:C, the ligand of TLR3, and LPS, the ligand of TLR4. Incubation with poly I:C (25 μg/ml) for 4 hours decreased the percentage of HCE cells staining positive for TLR3 from 11.84 ± 1.77% to 3.04 ± 2.37% (P < 0.05, Figure 3A). Following incubation with LPS (1 μg/ml) for 4 hours the percentage of HCE cells staining positive for TLR4 decreased from 17.31 ± 1.88% to 4.79 ± 2.37% (P < 0.05, Figure 3B).

HCF cells were likewise treated with either poly I:C or LPS, and the surface expression of TLR3 and TLR4, respectively, was evaluated using flow cytometry. Following stimulation using poly I:C, the surface staining of TLR3 decreased from 9.07% to 7.29% (P > 0.05). TLR4 activation using LPS resulted in decreased surface staining from 4.58% to 3.99% (P > 0.05). Hence, HCF stimulation using TLR-specific ligands did not result in a significant decrease in surface staining of the respective receptors.

### TLR3 and TLR4-specific mRNA levels are affected by receptor stimulation

**Figure 4:**
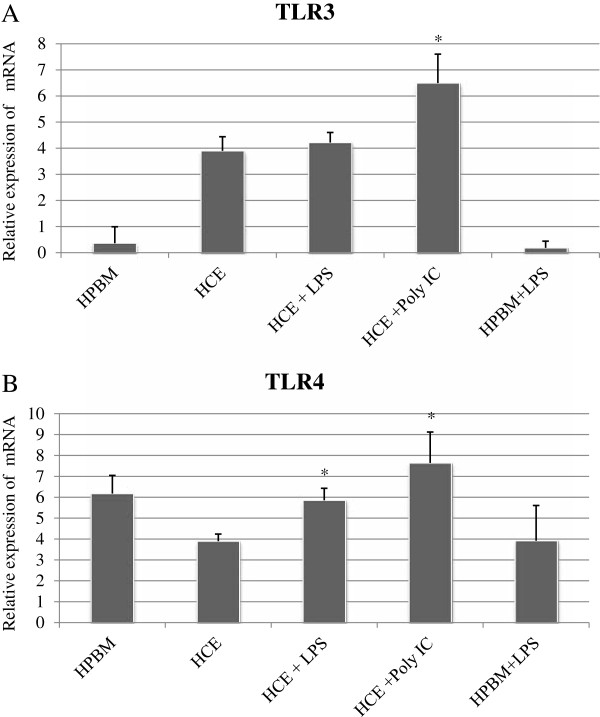
**TLR3 and TLR4-specific mRNA levels are affected by receptor stimulation.** Relative expression of TLR3-specific mRNA in naïve cells, and in cells treated with poly I:C in comparison to medium **(A)**. Relative expression of TLR4-specific mRNA in naïve cells and in cells treated with LPS in comparison to medium **(B)**. Data derived from four independent experiments. The asterisk (p < 0.05) represent statistical significance for experiments. All values are expressed as mean ± SD. TLR: Toll-like receptors, Human peripheral blood mononuclear, HCE: Human corneal epithelium, LPS: Lipopolysaccharide, Poly I:C: polyinosinic:polycytidylic acid.

HCE cells activation with poly I:C (Figure 4A) elicited an increase up to 6.49 ± 1.1 in the content of TLR3-specific mRNA in comparison to medium (P < 0.05). As expected, exposure of HCE cells to LPS did not elicit a statistically significant increase in TLR3-specific mRNA levels (P > 0.05). HCE cells activation with LPS (Figure 4B) elicited an increase up to 5.85 ± 0.57 in the content of TLR4-specific mRNA in comparison to medium (P < 0.05). Interestingly, HCE cells exposure to poly I:C elicited an increase in TLR4-specific mRNA which was statistically significant (P < 0.05; Figure 4B).

### Cytokines protein secretion in response to activation of HCE cells with LPS and poly I:C

**Figure 5:**
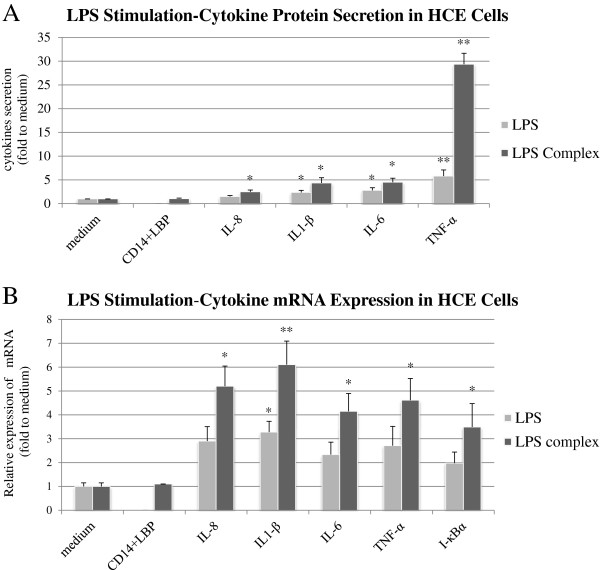
**HCE cells response to LPS and LPS complex (LPS combined with CD14 and LBP) induction.** The protein content of pro-inflammatory cytokines was measured using cytometric bead array **(A)**. Cytokine-specific mRNA content was measured using real-time PCR **(B)**. The data shown are representative of triplicate experiments. All values are expressed as mean ± SD. The asterisk (p < 0.05), double asterisk (p < 0.01) and three asterisks (p < 0.001) represent statistical significance for experiments as compare to cells incubated in medium alone. LPS: Lipopolysaccharide, LBP: Lipopolysaccharide binding protein, CD14: Cluster of differentiation.

**Figure 6:**
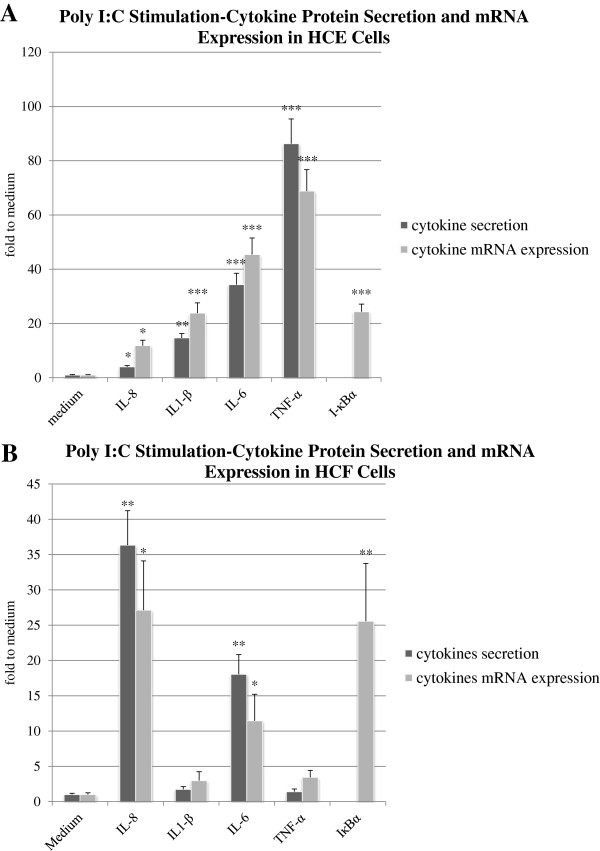
**HCE and HCF cells following activation with poly I:C and LPS respectively. A**. HCE cells response to poly I:C stimulation. HCE cells were incubated with poly I:C. The protein content of pro-inflammatory cytokines was measured using cytometric bead array technique **(A)**. The cytokine-specific mRNA content was measured using real-time PCR **(B)**. The data shown are representative of triplicate experiments. All values are expressed as mean ± SD. The asterisk (p < 0.05), double asterisk (p < 0.01) and three asterisks (p < 0.001) represent statistical significance for experiments as compare to cells incubated in medium alone. **B**. HCF cells response to LPS stimulation. The protein content of pro-inflammatory cytokines was measured using cytometric bead array technique (Figure 7A). Cytokine-specific mRNA content was measured using real-time PCR. The data shown are representative of triplicate experiments (Figure 7B). All values are expressed as mean ± SD. The asterisk (p < 0.05) and the double asterisk (p < 0.01) represent statistical significance for experiments as compare to medium.

Incubation of HCE cells with LPS complex (Figure 5A) and Poly I:C (Figure 6A) elicited higher levels of cytokines compared to cells incubated in medium alone.

Data from multiplex FBI demonstrated that the incubation of HCE cells with the LPS-alone elicited up to 2.8 ± 0.55-fold higher levels of IL-6 (P < 0.05), 1.5 ± 0.21-fold IL-8 (P > 0.05), 2.35 ± 0.46-fold IL-1β (P < 0.05) and 5.8 ± 1.3-fold TNFα (P < 0.01) compared to cells incubated in medium alone (Figure 5A). LPS stimulation combined with CD14 and LBP (LPS complex) increased cytokines production in HCE cells in relation to incubation with LPS alone in cell culture. The incubation of HCE with the LPS complex elicited up to 4.51 ± 0.85-fold higher levels of IL-6 (P < 0.05), 2.5 ± 0.36-fold IL-8 (P < 0.05), 4.35 ± 1.12-fold IL-1β (P < 0.05) and 29.35 ± 2.3-fold TNFα (P < 0.01) compared to cells incubated in medium alone (Figure 5A).

Incubation of HCE cells with the poly I:C elicited up to 34.31 ± 4.2-fold higher levels of IL-6 (P < 0.001), 4 ± 0.5-fold IL-8 (P < 0.05), 14.68 ± 1.65-fold IL-1β (P < 0.01) and 86.28 ± 9.1-fold TNFα (P < 0.001) compared to cells incubated in medium alone (Figure 6A).

### Cytokines mRNA expression in response to activation of HCE cells with LPS and poly I:C

The mRNA expression levels of IL-6, IL-1β, TNF-α and IL-8 were significantly increased in HCE cells upon stimulation with LPS complex (Figure 5B) and Poly I:C (Figure 6A) compared to non-stimulated cells.

Stimulation with LPS complex induced a significant elevation of the mRNA expression levels of IL-6 to 4.15 ± 0.52 fold (P < 0.05), IL-8 to 5.2 ± 0.6-fold (P < 0.05), IL-1β to 6.1 ± 0.45 fold (P < 0.01) and TNFα to 4.61 ± 0.8 fold (P < 0.05) compared to cells incubated in medium alone (Figure 5B).

Poly I:C stimulation induced a significant elevation of the mRNA expression levels of IL-6 to 45.41 ± 6.1fold (P < 0.001), IL-8 to 11.79 ± 2.05 fold (P < 0.05), IL-1β to 23.81 ± 3.8 fold (P < 0.001) and TNFα to 68.8 ± 7.9 fold (P < 0.001) compared to cells incubated in medium alone (Figure 6A).

### Cytokines protein secretion in response to activation of HCF with LPS and poly I:C

We found that LPS stimulation elicited both higher protein and mRNA contents of IL-6 and IL-8, as compared to cells incubated in medium alone.

**Figure 7:**
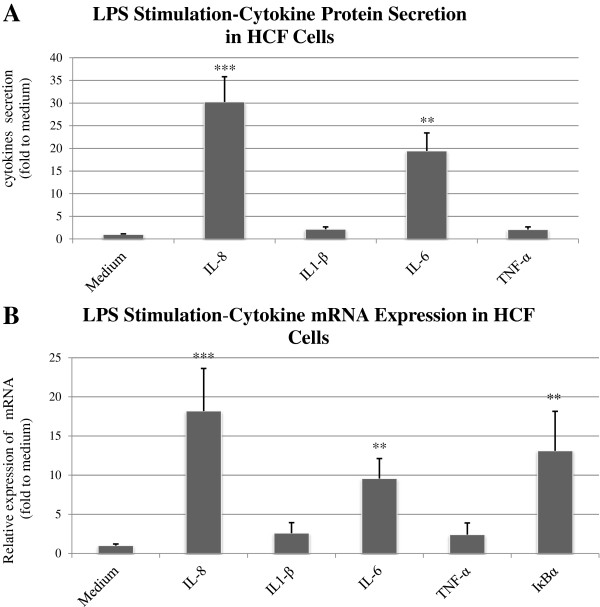
**HCF cells response to poly I:C stimulation.** HCF cells were incubated with poly I:C (5 μg/ml). The protein content of pro-inflammatory cytokines was measured using cytometric bead array technique **(A)**. The cytokine-specific mRNA content was measured using real-time PCR **(B)**. The data shown are representative of triplicate experiments. All values are expressed as mean ± SD. The double asterisk (p < 0.01) and three asterisks (p < 0.001) represent statistical significance for experiments as compare to medium.

Data from multiplex FBI demonstrate that the incubation of HCF cells with the LPS-only elicited up to 19.43 ± 3.99-fold higher levels of IL-6 (P < 0.01), 30.25 ± 5.58-fold IL-8 (P < 0.001), 2.14 ± 0.53-fold IL-1β (P > 0.05) and 2.09 ± 0.56-fold TNFα (P > 0.05) compared to cells incubated in medium alone (Figure 7A). Incubation of HCF cells with the Poly I:C elicited up to 18.03 ± 2.79-fold higher levels of IL-6 (P < 0.01), 36.32 ± 4.89-fold IL-8 (P < 0.01), 1.74 ± 0.38-fold IL-1β (P > 0.05) and 1.4 ± 0.39-fold TNFα (P > 0.05) compared to cells incubated in medium alone (Figure 6B).

Interestingly, we did not find an increased protein content of neither IL-1β nor TNF-α following LPS or poly I:C stimulation (Figures 7A and 6B).

### Cytokines mRNA expression in response to activation HCF cells with LPS and poly I:C

We found that stimulation of HCF cells using poly I:C elicited up to 11.45 ± 3.75 fold increase (P < 0.05) in IL-6 mRNA and 27.13 ± 6.97 fold increase in IL-8 mRNA levels (P < 0.05) as compared with to cells incubated in medium alone (Figure 6B).

Incubation of HCF cells with LPS elicited IL-6 mRNA up to 9.56 ± 2.56 fold higher (P < 0.01) and IL-8 mRNA levels to 18.19 ± 5.44 fold higher of (P < 0.001) compared to cells incubated in medium alone (Figure 7B).

Similar to the protein content results, the mRNA expression of neither IL-1β nor TNF-α were significantly elevated following LPS or poly I:C stimulation (Figures 7B and 6B).

### IκBα mRNA expression in response to activation HCE and HCF cells with LPS and poly I:C

The HCE cells stimulation with either LPS complex or Poly I:C elicited a significant elevation of IκBα mRNA expression levels to 3.49 ± 0.46 fold (P < 0.05, Figure 5B) and 24.32 ± 2.8 fold (P < 0.001, Figure 6A) respectively.

Incubation of HCF cells with either LPS complex or Poly I:C elicited a significant elevation of IκBα mRNA expression levels to 13.11 ± 5.04 fold (P < 0.01, Figure 7B) and 25.56 ± 8.18 fold (P < 0.01, Figure 6B) respectively.

## Discussion

In this study we brought evidence that HCE and HCF cells constitutively express mRNA specific for TLR2-4, TLR7 and TLR9 as well as TLR3- and TLR4- proteins on the cell surface. Our results suggest that these cells are capable of responding to TLR stimulation, culminating in the secretion of several pro-inflammatory mediators that are known to be down-stream effectors of the TLR pathway, along with up-regulation of their respective genes.

Our results show that while stimulation of HCE cells with LPS culminated in increased mRNA contents of both TLR3 and TLR4, HCE cells’ activation with poly I:C elicited increase mRNA levels of TLR3 only. This apparent inconsistency might be explained by a possible difference in the kinetics and half-lives of the different RNAs. A real-time PCR analysis conducted over a longer time-frame might counteract these differences.

Interestingly, activation of HCE TLR3 and TLR4 by poly I:C and LPS, while stimulating secretion of pro-inflammatory cytokines and the up-regulation of their respective genes, was found to decrease TLRs surface expression, as measured using flow cytometry. LPS-induced TLR4 suppression is an established concept. It has been described since the 1940s, that low doses of LPS can suppress the immune response to endotoxin challenge, a phenomenon termed LPS hyporesponsiveness or LPS tolerance [17-19]. The discovery of the Toll-like receptor family set a new interest in this phenomenon, and several mechanisms have been proposed to explain it, among them a decrease in surface expression of TLR4. The LPS tolerance might lead to better outcomes in patients with septic shock, perhaps by inhibiting the detrimental effects in an unchecked inflammatory process [20-22] Tolerance induction following TLR activation was previously observed in the human cornea. Kumar *et al*. observed that pre-exposure of HCE cells to low-dose flagellin, the ligand of TLR5, induced a state of tolerance, characterized by impaired functionality of the NF-κB, p38 and JNK pathways [23].

We hypothesize that the decrease in TLR3 and TLR4 surface expression in HCE cells following ligand activation is a generalization of the LPS tolerance described in the previous paragraph, and constitutes a negative-feedback loop that is important in containing the immune response, and might play a role in forming an immune-silent environment in the human cornea.

We observed that stimulation of TLR3 and TLR4 was followed by an increase in the content of TLR-specific mRNA. Interestingly, in apparent dissociation, the receptors’ protein content was noted to decrease. Our present findings are in accordance with a previous work on TLR4 expression in patients with sepsis, that revealed early TLR4-protein down-regulation, using flow cytometry. *Skinner et al*. postulated that these patients may at first respond with TLR4 activation and up-regulation, as shown by cytokine secretion profile, thereby over-expressing the pro-inflammatory cytokines that are characteristic of sepsis [24]. A subsequent TLR4 down-regulation could reflect a possible negative feedback mechanism, important for mitigating the inflammatory process. We postulate that a similar mechanism is found in HCE cells of the human eye. It is reasonable to assume, that while inappropriate secretion of pro-inflammatory cytokines is at best to be avoided and thus silenced, upon stimulation of TLR3 and TLR4, a preparatory phase, involving enhanced transcription of TLR3 and TLR4-specific genes, is beneficent. We must stress, nonetheless, that this last proposition is a mere hypothesis that has to be investigated in a further work.

Stimulation of TLR3 and TLR4 in HCF did not elicit a statistically significant decrease in surface expression, as was noted in HCE cells. It is interesting to note, that unlike HCE cells that responded with profound secretion of pro-inflammatory cytokines following TLR activation, HCF secreted only IL-6 and IL-8. These findings might reflect a distinct behavior of HCF, which may be more resistant to TLR stimulation, and less prone to initiate a profound inflammatory response.

## Conclusions

This work brought the first evidence for the expression of functional TLR3 and TLR4 receptors on human conjunctiva fibroblasts (HCF), capable of responding to TLR-specific ligands, and initiating an immune response by up-regulating pro-inflammatory genes and secreting various cytokines and inflammatory mediators. The data shown in this work may support the existence in the human cornea of the long-described LPS tolerance, and could reflect a protective mechanism against the inflammatory response. Along with the description of flagellin-induced TLR5 inhibition in the cornea, our data of TLR3 and TLR4 suppression by poly I:C and LPS could reflect a broader behavior of the TLR family in response to ligand stimulation, and help elucidate the nature of TLR-associated diseases. The HCF cells, important contributors to several ocular pathologies, were, as far as our research of the literature, devoid of a systematic characterization of the TLR family. This work has shown the gene and protein expression of several TLRs in HCF, and brought evidence to their functionality. Further research will help unraveling role of TLRs in conjunctival diseases.

## Abbreviations

TLRs: Toll-like receptors; HCE: Human corneal epithelial; HCF: Human conjunctival fibroblasts; LPS: Lipopolysaccharide; Poly I:C: Polyriboinosinic:polyribocytidylic acids; TNF-α: Tumor necrosis factor-α; IL: Interleukin; FBI: Fluorescent bead immunoassay; I-κBα: Inhibitory factor-κBα.

## Competing interests

The authors declared that they have no competing interests.

## Authors’ contributions

NE designed the study, performed the experiments, analyzed the data and wrote the manuscript. GA performed several experiments, wrote the manuscript and analysed data and statistics. EM performed several experiments, participated in the design of the study and in the analysis of the data. IA, CY and HM participated in the design of the study and provided material and expertise. HO designed the study, performed the experiments, analyzed the data and wrote the manuscript. AS designed the study, performed the experiments, analyzed the data and wrote the manuscript. All the experiments were performed in AS’s and HO’s laboratories. All authors read and approved the final manuscript.
